# Lead in Spices, Herbal Remedies, and Ceremonial Powders Sampled from Home Investigations for Children with Elevated Blood Lead Levels — North Carolina, 2011–2018

**DOI:** 10.15585/mmwr.mm6746a2

**Published:** 2018-11-23

**Authors:** Kim A. Angelon-Gaetz, Christen Klaus, Ezan A. Chaudhry, Deidre K. Bean

**Affiliations:** ^1^North Carolina Department of Health and Human Services; ^2^Wake County Environmental Services, Raleigh, North Carolina; ^3^University of North Carolina at Chapel Hill; ^4^Kansas State University, Manhattan, Kansas.

The number of pediatric cases of elevated blood lead levels (BLLs) are decreasing in North Carolina. However, one county reported an increase in the number of children with confirmed BLLs ≥5 *μ*g/dL (CDC reference value, https://www.cdc.gov/nceh/lead/acclpp/blood_lead_levels.htm), from 27 in 2013 to 44 in 2017. Many children with elevated BLLs in this county lived in new housing, but samples of spices, herbal remedies, and ceremonial powders from their homes contained high levels of lead. Children with chronic lead exposure might suffer developmental delays and behavioral problems (https://www.cdc.gov/nceh/lead/). In 1978, lead was banned from house paint in the United States (*1*); however, children might consume spices and herbal remedies daily. To describe the problem of lead in spices, herbal remedies, and ceremonial powders, the North Carolina Childhood Lead Poisoning Prevention Program (NCCLPPP) retrospectively examined properties where spices, herbal remedies, and ceremonial powders were sampled that were investigated during January 2011–January 2018, in response to confirmed elevated BLLs among children. NCCLPPP identified 59 properties (6.0% of all 983 properties where home lead investigations had been conducted) that were investigated in response to elevated BLLs in 61 children. More than one fourth (28.8%) of the spices, herbal remedies, and ceremonial powders sampled from these homes contained ≥1 mg/kg lead. NCCLPPP developed a survey to measure child-specific consumption of these products and record product details for reporting to the Food and Drug Administration (FDA). Lead contamination of spices, herbal remedies, and ceremonial powders might represent an important route of childhood lead exposure, highlighting the need to increase product safety. Setting a national maximum allowable limit for lead in spices and herbal remedies might further reduce the risk for lead exposure from these substances.

All BLLs for North Carolina children aged <6 years are required to be reported to NCCLPPP, along with demographic data including race and Hispanic ethnicity of the child. Approximately 51% of North Carolina children are tested during routine well child visits at age 1 or 2 years. Diagnostic testing of a second (preferably venous) blood specimen at a reference laboratory is required to confirm all BLLs ≥5 *μ*g/dL. Since July 1, 2017, a confirmed elevated BLL has been defined in North Carolina as two consecutive test results ≥5 *μ*g/dL within a 12-month period; previously, a confirmed elevated BLL was defined as two consecutive test results ≥10 *μ*g/dL within a 6-month period.*

Lead investigators from state and local health departments offer free home investigations for children with confirmed elevated BLLs. Because of the lag time between the initial and diagnostic specimen collection and laboratory result reporting, investigations might be scheduled several months after the initial BLL specimen was collected. During January 2011–January 2018, home lead investigations were conducted at 983 properties in North Carolina. Lead investigators collected information on when the home was built, documented any evidence of lead in the home, and submitted environmental samples of lead paint, water, soil, consumer products, and foods to the North Carolina State Laboratory of Public Health for chemical analysis. All spice, herbal remedy, and ceremonial powder samples were screened for lead with an atomic absorption mass spectrometer by the North Carolina State Laboratory of Public Health; starting in 2011, samples with <15 mg/kg of lead were subsequently analyzed using an inductively coupled plasma mass spectrometer. Lead investigators entered investigation reports and environmental sample data into the North Carolina childhood lead surveillance system and linked investigations to the children’s blood lead test results.

For the environmental samples tested, results below the limit of detection (LOD) were replaced by LOD divided by √2, an extrapolation technique with low error rates (*2*). Blood lead test results below LOD were standardized to 1 *μ*g/dL. Because information about consumption was not collected for most children, descriptive statistics were calculated for the environmental sample results separately from the child blood lead data. The data analysis and figures for this paper were generated using statistical software.

Among the 61 children included in this report, the average screening (initial) BLL was 17.0 (±9.6) *μ*g/dL (Figure), and the average diagnostic BLL was 15.2 (±7.0) *μ*g/dL. Diagnostic BLLs were drawn between February 28, 2011, and December 5, 2017. The average age of the children at the time of the lead investigation was 2.3 years (range = 0.9 to 6.6 years); investigations for these children were conducted from March 17, 2011, to January 26, 2018.

**FIGURE:**
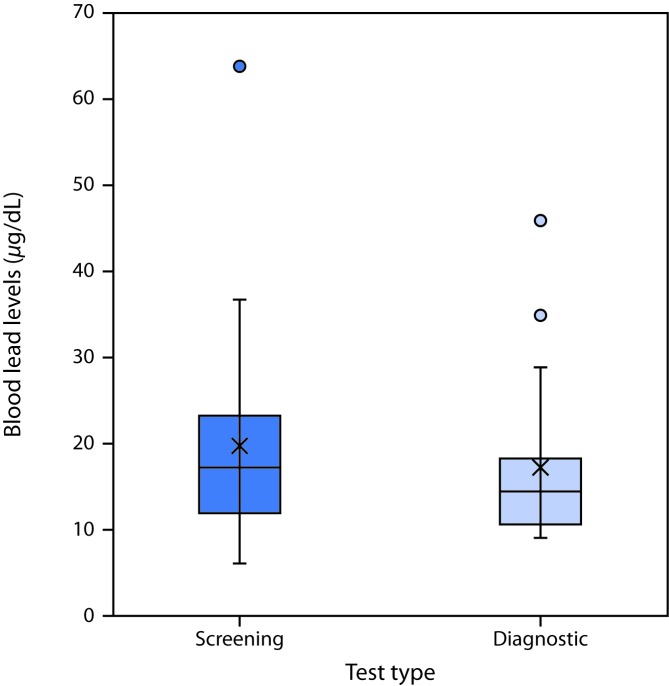
Screening and diagnostic* blood lead levels in children (n = 61) exposed to lead-contaminated spices, ceremonial powders, or herbal remedies — North Carolina, 2011–2017 * Box plots illustrate the distributions of screening (initial) and diagnostic (confirmatory) blood lead levels. The tops of the boxes represent the 75th percentile and the bottoms the 25th percentile. The middle line of the box is the median. X represents the mean. Circles represent outliers. Whiskers indicate the standard deviations.

Information on race was available for 58 (95%) children. Among those with known race, 41 children (67.2%) were identified as Asian (including those of Indian and Pakistani descent); nine children (13%) were identified as black or African American (including two siblings born in West Africa). Among eight children identified as white, one was from Afghanistan. Among 51 children (84%) for whom Hispanic ethnicity was known, seven (11.5%) were Hispanic.

The 59 properties investigated were in eight primarily urban counties; 42 properties (71%) were built after 1978. Among these 42 newer residences, 10 had brass objects, jewelry, cookware, and other consumer items that might have contained lead; 32 (76%) had no evidence of lead in paint, dust, miniblinds, faucets, bathtub glaze, or furniture finish. In seven of these 32 properties, spices, herbal remedies, and ceremonial powders were the only identified risk.

A total of 392 samples of spices, herbal remedies, and ceremonial powders were collected from the 59 properties. Six sample results were excluded because of different sampling and analysis methods. Among the remaining 386 samples included in this report, 344 (89%) were items intended for consumption (food), including spices and herbal remedies; and 42 (11%) were items not intended for consumption (nonfood), including ceremonial powders. Mean lead levels of ≥1 mg/kg were identified in 50 product categories, including 10 nonfood categories and 40 food categories. Among 177 samples included in these 50 product categories, 111 (62.7 %) individual samples were contaminated with ≥1 mg/kg lead, including 76 (22.0%) food items and 35 (83.3%) nonfood items (Table). These 111 contaminated samples represent 28.8% of the 386 samples included. Among nonfood items (ceremonial powders and topical remedies), the highest average lead levels were detected in kumkum (average = 12,185 mg/kg; range = 0.4–140,000), sindoor (average = 41,401 mg/kg; range = 0.1–130,000), and surma (average = 68,000 mg/kg; only one sample collected). Among edible items, saffron supplement (average = 2,764 mg/kg; only one sample collected), Balguti Kesaria (an Ayurvedic medicine) (average = 220 mg/kg; only one sample collected), and turmeric (average = 66 mg/kg; range = 0.1–740) had the highest average lead levels. Country of purchase was recorded for 187 (48%) of the 386 samples; therefore, product origin is largely unknown. Among samples with known origin, 142 (76%) were purchased in the United States.

**TABLE:** Categories of spices, herbal remedies, and ceremonial powders (N = 177) with average lead level ≥1 mg/kg sampled during lead investigations — North Carolina, 2011–2018

Product category	No. of samples	Average lead level, mg/kg (SD)	Range, mg/kg
**Nonfood items**			
Ash powder	1	19.0 (N/A)	N/A
Incense	4	7.0 (6.6)	1.9–15.7
Kumkum (powder made from turmeric or other materials, used for social and religious markings in India)	12	12,185.2 (40,276.5)	0.4–140,000.0
Pooja powder (used in Hindu religious worship)	1	65.0 (N/A)	N/A
Rangoli (colored powders used to make designs)	2	2.9 (1.8)	1.6–4.2
Sandal scented pooja powder	2	4.2 (1.6)	3.0–5.3
Sandalwood (chandan) powder	3	8.4 (9.2)	3.0–19.0
Sindoor (traditional red cosmetic powder)	8	41,401.1 (58,540.7)	0.1–130,000.0
Surma (an ore ground into powder, used as an eye cosmetic)	1	68,000.0 (N/A)	N/A
Vibhuti (ash made from burnt dried wood, applied to the skin in religious rituals)	3	80.3 (70.2)	2.9–140.0
**Food items**			
**Spices and condiments**			
Anise	4	1.7 (1.9)	0.3–4.4
Bay leaves	1	2.6 (N/A)	N/A
Black seeds	1	2.6 (N/A)	N/A
Cardamom	1	1.4 (N/A)	N/A
Chaat masala	1	1.5 (N/A)	N/A
Chili garlic sauce	1	4.0 (N/A)	N/A
Chili powder/Red pepper	23	12.6 (41.2)	0.1–170.0
Cinnamon	2	2.6 (0.1)	2.5–2.7
Cloves	1	1.4 (N/A)	N/A
Coriander	9	4.8 (12.8)	0.1–39.0
Cumin	17	1.1 (1.5)	0.1–6.4
Cumin and coriander mix	2	1.1 (0.5)	0.7–1.4
Curry leaf powder	1	1.4 (N/A)	N/A
Curry powder	2	1.4 (1.7)	0.2–2.6
Dagad phool (stone flower)	1	2.8 (N/A)	N/A
Fenugreek	1	1.4 (N/A)	N/A
Ginger	3	1.0 (0.5)	0.7–1.6
Lemon powder	1	6.5 (N/A)	N/A
Kabsa spice	1	19.0 (N/A)	N/A
Mint	1	2.0 (N/A)	N/A
Rosemary	1	1.6 (N/A)	N/A
Saffron	2	1.2 (1.4)	0.2–2.2
Shwarma spice	1	6.8 (N/A)	N/A
Spice mix (all purpose)	3	1.8 (2.6)	0.2–4.8
Turmeric	34	66.4 (206.6)	0.1–740.0
Vanilla	1	8.5 (N/A)	N/A
**Medications, oils, and supplements**			
Balguti Kesaria (Ayurvedic medicine)	1	220.0 (N/A)	N/A
Chamomile oil	1	8.2 (N/A)	N/A
Herbal remedy	1	8.2 (N/A)	N/A
Lime calcium powder	1	1.4 (N/A)	1.4
Nux vomica	1	10.6 (N/A)	N/A
Mojhat ceremonial drink	1	31.0 (N/A)	N/A
Saffron supplement	1	2,764.0 (N/A)	N/A
**Prepared foods**			
Candy	5	10.6 (14.0)	0.0–25.9
Milk cookie	1	1.4 (N/A)	N/A
**Other food products**			
Baby cereal	2	17.6 (23.2)	1.2–34.0
Cornstarch	2	5.4 (6. 6)	0.7–10.0
Rice flour	3	4.1 (5.7)	0.1–10.6
Rice with turmeric	1	1.4 (N/A)	N/A
Sugar	3	3.7 (6.0)	0.1–10.6

**Abbreviations:** N/A = not applicable; SD = standard deviation.

## Discussion

Lead can contaminate spices during many points in the global supply chain. Spices are often grown in countries polluted by leaded gasoline, smelters, battery manufacturing plants, and mines. Lead is deposited in soil and water from airborne pollutants and fertilizer application. Lead dust from grinding machinery can also contaminate spices (*3*). Spices might also be adulterated deliberately with lead to enhance color or increase weight.^†^ Because >95% of spices consumed in the United States are imported,^§^ recommendations to purchase only locally grown spices are impractical. According to the World Health Organization Codex Standard 193–1995, the permissible limit of lead for infant formula is 0.02 mg/kg lead and for salt is 2 mg/kg. No U.S. permissible limit for lead in spices exists; however, the FDA limit for lead in natural-source food color additives (e.g., paprika, saffron, and turmeric) is 10 mg/kg. The FDA action levels (i.e., the levels at which an investigation is undertaken, or a recall is issued, depending upon the circumstances and findings) for products intended for consumption by children are 0.1 mg/kg for candy and 0.5 mg/kg for other foods^¶^; however, spices are not considered food intended for consumption by children. The Environmental Protection Agency estimates of consumption from the What We Eat in America survey are low for many of the spices in question (e.g., 0.09 g/day of cumin, 0.03 g/day of turmeric) (*4*); however, spice consumption might differ for children whose parents emigrated from Southeast Asia (e.g., estimated consumption: 1.22 ± 1.14 g per portion of cumin in dishes prepared daily; 0.60 ± 0.46 g per portion of turmeric in dishes prepared daily), where spices are used in cooking, home remedies, and ceremonial activities (*5*). Use of spices, herbal remedies, and alternative medicines also are increasingly popular among other U.S. residents; spice imports into the United States have increased by approximately 50% since 1998 (*6*). However, their regulation is complicated by Internet sales, international travel, and importation by relatives and friends (*7*).

A large proportion of ceremonial powders, spices, and herbal remedies found during home investigations for children with elevated BLLs in North Carolina were contaminated with lead. Spices and herbal remedies are meant for consumption, used to enhance food flavor and color, and are administered medicinally to persons of all ages. Lead investigators reported that spices and herbal remedies are used by both recent immigrants and U.S.-born children. Although ceremonial powders are not food, they might be accidentally ingested by children.

Most previous reports of childhood lead poisoning from spices are case reports (*7*,*8*). This study includes approximately 7 years of data, environmental investigation results, and clinical findings from 61 children for whom these substances were a suspected source of lead exposure.

Because the level of detail reported on spice sample consumption and product origin was inconsistent among lead investigators, NCCLPPP created a survey tool to guide and encourage lead investigators to collect the details necessary for FDA reporting. This survey tool was piloted during home investigations in one North Carolina county. The survey also was tested for cultural sensitivity, administration time, and ease of understanding through focus groups with Hispanic and South Asian community members. The survey tool is available online in English** and Spanish.^††^

New York City, New York State, and California have created their own recall and alert protocols for contaminated products (*9*,*10*). NCCLPPP leads a quarterly, national workgroup to develop standardized protocols for product reporting and data and sample collection. To reduce the time for reporting to FDA, NCCLPPP added a new workflow to the North Carolina childhood lead surveillance system, which lists new lead poisoning cases from consumable items. If the spices or herbal remedies are purchased in the United States, the NCCLPPP epidemiologist will notify the FDA Consumer Safety Officer regional liaison of the findings. In 2017, FDA formed a Toxic Elements Working Group to focus on protecting consumers from heavy metals such as lead in food, cosmetics, and dietary supplements (https://www.fda.gov/Food/FoodborneIllnessContaminants/Metals/ucm604173.htm).

The findings in this report are subject to at least five limitations. First, spices are frequently purchased wholesale and removed from their original containers, so information regarding product origins and lot numbers might have been discarded. Second, many lead investigators did not collect spice and herbal remedy samples. The authors excluded 11 reports from the numerator but not the denominator of the analysis (all lead investigations since January 1, 2011), in which cultural products were not sampled, although they were suspected as a lead exposure hazard, so the number of cases with exposure to these products might be underestimated. Third, until recently, persons with BLLs confirmed between 5–9 µg/dL were only offered education and clinical management unless they lived in a county with a local ordinance triggering home investigation at lower levels than the state guidelines, which also may lead to an underestimation of cases. Fourth, although some individual specimens contained low detectable lead levels, the combined, chronic exposure to these products might increase BLLs in some children. Direct toxicologic modeling cannot be performed using these data because of the large amount of missing information regarding consumption. Finally, the small sample size and the large age range of children would make modeling the effects of these exposures difficult because the metabolism of lead and effect of lead on the development of an infant aged 1 year would be different from that for a child aged 5 years.

Lead poisoning prevention professionals should educate parents about the potential for lead exposure from spices, herbal remedies, and ceremonial powders by making educational materials available in several languages at festivals, places of worship, and other community centers. Keeping ceremonial powders out of reach of children can prevent their accidental consumption, and testing of children who consume spices or herbal remedies regularly might lead to earlier detection of elevated BLLs (https://www.cdc.gov/nceh/lead/tips/folkmedicine.htm). Lead investigators should sample these products during investigations and attempt to document product origin and level of consumption. Increasing testing of spices, herbal remedies, and ceremonial powders for heavy metals by food safety regulators at the port of entry when these substances are imported into the United States might reduce the occurrence of lead poisoning associated with these substances.^§§^ Because these products are sold nationwide, setting a national maximum allowable limit for lead in spices and herbal remedies might further reduce the risk for lead exposure from them.

**Summary**

**What is already known about this topic?**

No national limit exists for lead contamination in spices. Ingested lead is absorbed quickly by children and causes developmental delays.

**What is added by this report?**

A North Carolina study of lead content in spices, herbal remedies, and ceremonial powders in homes of children with elevated blood lead levels found that 28.8% of samples contained ≥1 mg/kg lead, suggesting contaminated products might represent an important source of childhood lead exposure. A survey instrument was created to collect information on product origin and consumption.

**What are the implications for public health practice?**

Spices and herbal remedies are increasingly part of U.S. children’s diets and might be a source of lead exposure in children with elevated blood lead levels.

## Footnotes

* Public Health Law of North Carolina §130A-131.5-131.9H. https://www.ncleg.net/EnactedLegislation/Statutes/PDF/ByChapter/Chapter_130A.pdf.

† https://www.astaspice.org/the-american-spice-trade-associations-statement-on-lead-in-turmeric/.

§ https://www.ers.usda.gov/data-products/chart-gallery/gallery/chart-detail/?chartId=58398.

¶ https://www.fda.gov/food/foodborneillnesscontaminants/metals/ucm172050.htm.

** https://ehs.ncpublichealth.com/docs/forms/cehu/SpiceandHomeRemedySurveyFINAL-English.pdf.

†† https://ehs.ncpublichealth.com/docs/forms/cehu/SpiceandHomeRemedySurveyFINAL-Spanish.pdf.

§§ https://www.fda.gov/food/guidanceregulation/importsexports/importing/ and https://www.fda.gov/Food/FoodborneIllnessContaminants/Metals/ucm2006791.htm.
